# TCQI-YOLOv5: A Terminal Crimping Quality Defect Detection Network

**DOI:** 10.3390/s25247498

**Published:** 2025-12-10

**Authors:** Yingjuan Yu, Dawei Ren, Lingwei Meng

**Affiliations:** School of Energy and Mining Engineering, Shandong University of Science and Technology, Qingdao 266590, China

**Keywords:** automotive industry, terminal crimping, YOLOv5, defect detection, attention mechanism

## Abstract

With the rapid development of the automotive industry, terminals—as critical components of wiring harnesses—play a pivotal role in ensuring the reliability and stability of signal transmission. At present, terminal crimping quality inspection (TCQI) primarily relies on manual visual examination, which suffers from low efficiency, high labor intensity, and susceptibility to missed detections. To address these challenges, this study proposes an improved YOLOv5-based model, TCQI-YOLOv5, designed to achieve efficient and accurate automatic detection of terminal crimping quality. In the feature extraction module, the model integrates the C2f structure, FasterNet module, and Efficient Multi-scale Attention (EMA) attention mechanism, enhancing its capability to identify small targets and subtle defects. Moreover, the SIOU loss function is employed to replace the traditional IOU, thereby improving the localization accuracy of predicted bounding boxes. Experimental results demonstrate that TCQI-YOLOv5 significantly improves recognition ccuracy for difficult-to-detect defects such as shallow insulation crimps, achieving a mean average precision (mAP) of 98.3%, outperforming comparative models. Furthermore, the detection speed meets the requirements of real-time industrial applications, indicating strong potential for practical deployment.

## 1. Introduction

The development of the automotive industry has driven the growth of the automotive parts sector. Terminals, as essential components of wiring harnesses, ensure stable and reliable signal transmission through a secure connection with electrical wires [1,2]. The crimping process is a crucial step in connecting terminals and wires, and with the increasing scale and automation of industrial production, issues concerning the quality of terminal crimping have become increasingly prominent. Therefore, the development of efficient Terminal Crimping Quality Inspection (TCQI) technologies is of vital importance [3,4].

Terminal crimping quality inspection, similar to other types of industrial defect detection, aims to identify defective products from a large number of items produced on the assembly line [5,6,7,8]. At present, terminal crimping quality inspection is still predominantly performed manually. This approach requires inspectors to visually assess the crimping quality of terminals over extended periods, while the production speed of modern assembly lines has far surpassed the capacity of manual inspection. As a result, manual inspection is both labor-intensive and inefficient. Therefore, there is an urgent need for an efficient, accurate, and fully automated detection method to overcome these limitations.

With the advancement of computer technology, machine vision–based methods have been increasingly applied in the field of defect detection. He et al. proposed an adaptive multi-scale defect detection method for wind turbine blade surfaces, which employs a multi-level feature extraction module and an adaptive bounding box proposal module to delineate multi-scale defect regions, followed by training a binary classifier to distinguish between defective and non-defective areas [9]. Feng et al. developed a YOLOv5-based defect detection algorithm for aluminum profiles that integrates attention mechanisms and multi-scale features, focusing on the feature information of defect regions within aluminum defect datasets to improve the detection performance for small targets [10]. Tang et al. proposed an enhanced chip surface defect detection algorithm based on an improved version of YOLOv8, which enhances the expressiveness of deep features and significantly improves the recognition accuracy of small defect targets [11]. Li et al. introduced a YOLOv4-based online metal surface defect detection method for wire arc additive manufacturing, in which the channel attention mechanism in the backbone network effectively improves detection performance. Additionally, the multi-SPP structure in the FPN extracts supplementary information with varying receptive field sizes, thereby enhancing mean average precision [12]. Zhang proposed a lightweight defect detection network designed for resource-constrained scenarios, combining a channel attention mechanism to extract finer defect features under complex background interference and improving the accuracy of small-defect detection. Moreover, the incorporation of Focal Loss mitigates the imbalance between the number of default boxes for defects and background regions during training [13]. Lu addressed the issue of low detection accuracy in X-ray weld defect detection using YOLOv8 by introducing an enhancement strategy. An additional micro-object detection head was added to the detection module, enabling more precise capture of extremely small defect features, effectively expanding the lower detection limit and significantly improving the model’s ability to detect minute weld defects [14]. Wang et al. proposed an autonomous identification framework for composite faults of mechanical equipment based on reinforcement adversarial open set algorithm, and used a spike neural network with continuous time-frequency gradient for bio-inspired compound defect detection. They also used multi-label domain adversarial reinforcement learning for unsupervised composite fault identification [15,16,17]. Peng et al. used a large language model to realize adaptive fault diagnosis of onboard controllers of railway vehicles [18].

Current defect detection models often exhibit poor generalization capabilities; when defect types are diverse, a single or simple model fails to achieve accurate identification. Moreover, the role of the loss function in defect detection is critical and should not be overlooked. In addition, there remains a research gap in the field of terminal crimping quality inspection, underscoring the urgent need for related studies to fill this gap.

To address the aforementioned issues, this study proposes a TCQI model based on YOLOv5, referred to as TCQI-YOLOv5. We chose YOLOv5 as our infrastructure because of its maturity, stability, lightweight design, and high efficiency. By specifically enhancing its feature extraction and localization capabilities, we constructed a TCQI-YOLOv5 model suitable for terminal crimping quality inspection. The main innovations of TCQI-YOLOv5 are as follows: (1) A novel C2f-fast-EMA hybrid module is designed to replace the original C3 structure in YOLOv5, significantly enhancing feature extraction for subtle terminal crimping defects. (2) It integrates a partially convolutional structure inspired by FasterNet, replacing standard convolutions with its partial convolution (PConv) operation in the bottleneck module. This design cleverly reduces computational cost and memory access, thereby improving processing speed without sacrificing accuracy, thus enhancing inference speed under industrial real-time constraints. (3) The SIOU loss is adopted to improve bounding-box regression by incorporating angle, distance, and shape factors, which is particularly beneficial for small, elongated defect regions. (4) A new TCQI dataset containing eight fine-grained terminal crimping defect categories is constructed, which has not been addressed in previous studies.

## 2. Data Source

### 2.1. Terminal Crimping Process and Quality Issues

**Figure 1:**
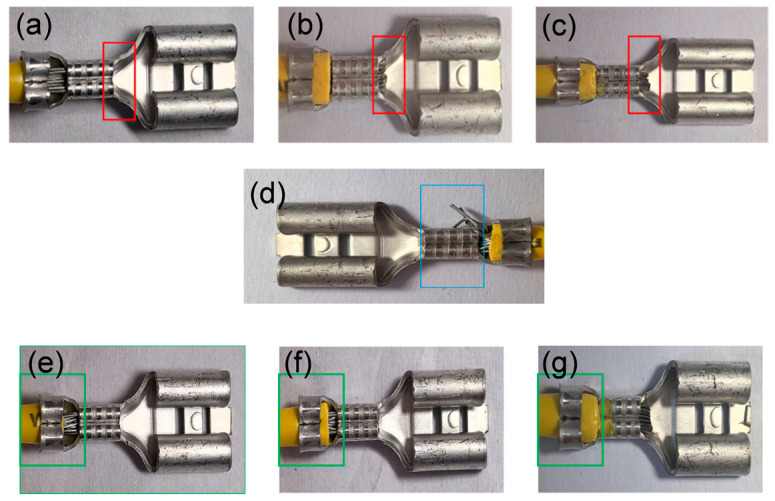
(**a**–**c**) correspond to the wire tip with shallow crimping, normal crimping, and deep crimping, respectively; (**d**) shows copper exposure at the crimp barrel; and figures (**e**–**g**) correspond to the insulation sleeve with shallow crimping, normal crimping, and deep crimping, respectively.

Terminal crimping involves the application of external force to securely bond the conductor core with the contact surface of the terminal. This force is primarily applied through a die, making the process a stamping operation, during which the terminal undergoes elastic–plastic deformation [19]. The crimping process directly determines the quality of the terminal connection; factors such as the terminal’s shape, crimping height, applied crimping force, and the specific crimping location all influence the final quality [20]. During wiring harness production, a large number of terminals must be crimped, and crimping defects are inevitably generated in the process. Based on the locations where defects occur after crimping the terminal and conductor, inspection is typically concentrated on three key areas: the wire tip, the crimp barrel, and the insulation sleeve. The wire tip refers to the exposed part of the conductor that should extend to an appropriate length. The crimp barrel is the area where the terminal directly connects with the conductor. The insulation sleeve is the section at the terminal tail that connects with the insulation; here, a portion of the insulation should be exposed while ensuring that it does not completely cover the conductor. The appearance of defects in these three terminal areas is shown in Figure 1, and the corresponding causes and morphologies of the defects are summarized in Table 1. All identified defect types are visually distinguishable.

In this study and the target industrial application, the quality of terminal crimping is evaluated according to established acceptance criteria. Crimping defects are classified based on the following principle: a pass/fail condition is considered acceptable only when all three inspected areas—the wire tip, crimp barrel, and insulation sleeve—are in a normal state. A defect is defined as any deviation from the normal state in any of the three areas. Based on Failure Mode and Effects Analysis (FMEA), detected defects are further categorized into two severity levels. Critical defects immediately cause product failure or safety hazards (e.g., short circuit, open circuit). Major defects primarily affect the long-term mechanical stability and reliability of the connection but may not immediately lead to failure.

### 2.2. Image Acquisition

The resolution of the image acquisition camera directly affects the results of quality inspection. Based on actual measurements, the terminal has a width of approximately 8 mm and a length of approximately 18 mm. However, during practical inspection, defect locations may not necessarily appear within the nominal terminal area. Therefore, when capturing images, the terminal’s deformation and the spatial relationship of potential defects are taken into account. A redundancy of 0.5 times the terminal length and 1 time the terminal width is reserved, resulting in a field of view with a length of 27 mm and a width of 16 mm. The minimum detectable defect size *N_min_* is 0.1 mm. The minimum defect size threshold of 0.1 mm was determined based on the industrial quality standards adopted by our manufacturing partner for terminal crimping. This value represents the smallest dimensional deviation that is considered critical enough to be classified as a functional defect, ensuring that the model focuses on identifying deviations that compromise product reliability. To enhance the stability of the terminal crimping quality inspection system, an accuracy of 5 pixels per unit is adopted, i.e., the precision coefficient *K* = 5 pixels/0.01 mm. The accuracy coefficient used in our system represents the physical resolution of the image acquisition device. This value is determined through a rigorous camera calibration process based on a specific industrial camera and lens configuration. Specifically, the system is calibrated so that one pixel corresponds to a physical size of approximately 0.002 mm on the object plane. This high resolution ensures that even the smallest features and deviations, whose dimensional tolerances in industry standards are typically on the order of one-hundredth of a millimeter, can be adequately represented by a sufficient number of pixels, thus guaranteeing the sensitivity and accuracy required to detect minute crimping defects. Accordingly, the horizontal resolution *K_L_* and vertical resolution *K_H_* of the camera are expressed as follows:

(1) $$K_{L}=\frac{L_{v}\times K}{N_{\min}}$$

(2) $$K_{H}=\frac{W_{v}\times K}{N_{\min}}$$

where *L_V_* represents the field of view length, set to 27 mm, and *W_V_* represents the field of view width, set to 16 mm. The Hikvision MV-CU060-10GC camera (Hikvision, Hangzhou, China) was selected as the image acquisition device, with its specific parameters listed in Table 2.

The acquired images were processed using denoising, histogram equalization, and color space transformation to improve the signal-to-noise ratio (SNR). For data augmentation, YOLOv5’s default data augmentation strategies were used, including Mosaic, random scaling, HSV gamut perturbation, horizontal flipping, and random cropping, to improve the model’s robustness to the complex environments of real-world terminal images. The TCQI categories are shown in Table 3. We set 500 images for each of the eight TCQI types, resulting in a total of 4000 images for the experiment. Then, Labelimg 1.8.6 software was used to annotate the header, pressure foot, and insulation of all TCQI categories.

## 3. Methodology

### 3.1. YOLOv5 Model and Its Limitations in Terminal Detection

YOLOv5 is a deep learning–based object detection algorithm developed by the Ultralytics team [21]. Compared with previous versions, YOLOv5 introduces several improvements and optimizations in both speed and accuracy. It employs CSPDarknet53 as the backbone network, integrating the features of Cross Stage Partial Network (CSP) and Darknet53 to enhance the network’s representational capacity. Its lightweight network architecture increases detection speed, allowing single-image detection times to range from tens to hundreds of milliseconds, making it particularly suitable for real-time detection tasks, such as those in this study [22].

In terminal crimping quality inspection, the YOLOv5 model achieves mean average precisions (mAP) of 89.4%, 90.1%, and 98.2% for shallow, normal, and deep insulation crimps, respectively, which are noticeably lower than those for the crimp barrel and wire tip regions (generally > 97%). The shallow insulation crimp, in particular, exhibits the lowest recognition accuracy, significantly affecting the overall reliability of detection. Moreover, the model’s feature extraction capability is limited. The original C3 module used in YOLOv5 has restricted ability in feature extraction and reuse, making it difficult to capture subtle differences in the insulation sleeve area. The absence of an attention mechanism for key regions, such as variations in insulation length, renders the model insensitive to fine defects. Additionally, bounding box localization shows deviations; the overlap between predicted and ground-truth boxes is suboptimal, especially for small targets like insulation edges [23,24]. The conventional IOU loss function fails to adequately reflect differences in angle, distance, and shape between predicted and actual boxes. Therefore, to address these issues, the model requires improvements to enhance the detection accuracy for insulation sleeves and meet the specific requirements of terminal crimping quality inspection.

### 3.2. Enhanced YOLOv5: TCQI-YOLOv5

**Figure 2:**
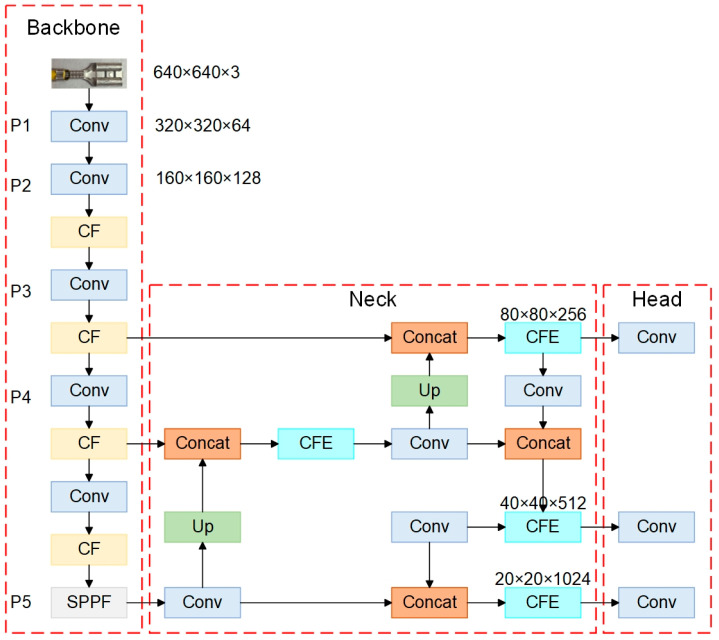
The improved model structure of TCQI-YOLOv5 (CF stands for C2f-Faster, and CFE stands for C2f-Faster-EMA).

To address these issues, we propose an enhanced YOLOv5-based model, termed TCQI-YOLOv5. The following strategies are adopted to improve detection performance: (1) Replacement of the original C3 module with a C2f-fast-EMA module to enhance the capability of the feature extraction backbone. (2) Utilization of SIOU in place of IOU to optimize the bounding box regression loss, thereby improving localization accuracy for terminal crimping quality inspection tasks. The improved TCQI-YOLOv5 model structure is shown in Figure 2.

#### 3.2.1. Improvement of Feature Extraction Module

The original C3 module in YOLOv5 exhibits insufficient accuracy for terminal crimping quality inspection tasks, particularly in detecting the insulation sheath component. To address this limitation, we replace the C3 module with the proposed C2f-fast-EMA module in the backbone network. It is worth noting that since the EMA mechanism demonstrates optimal effectiveness during image feature extraction in the backbone, the Neck part of our model employs the C2f-fast module instead of C2f-fast-EMA to achieve more efficient real-time detection of terminal crimping quality. The specific architectural improvements are detailed below.

(1) The C2f module first employs a CBS (Convolution-BatchNorm-SiLU) module to reduce the number of channels, which decreases computational load and memory usage during training, thereby facilitating model deployment [25]. Subsequently, a Split operation rearranges the feature maps along the channel dimension, followed by multiple convolutional operations for feature extraction. The Split and Concat operations effectively create residual connections that directly propagate input information to deeper layers, enhancing the accuracy of feature representation. Finally, a convolution layer restores the original channel size. This residual design mitigates the vanishing gradient problem inherent in the original network and optimizes the feature learning capability.

**Figure 3:**
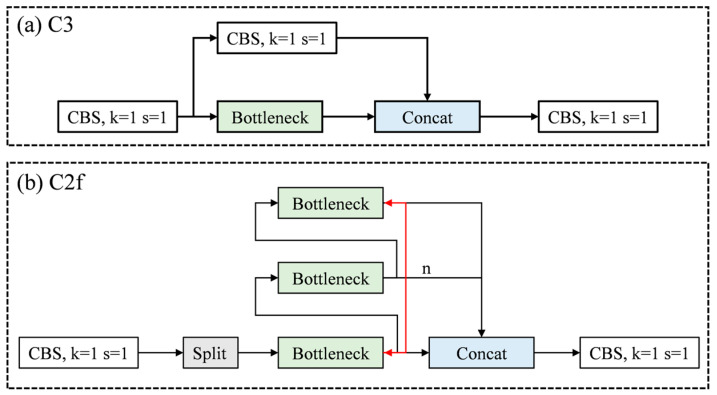
Schematic diagrams of the C3 and C2f modules.

In terminal crimping quality inspection, the objects of interest often occupy a relatively small portion of the image, making it a small-object detection task. The architecture of the C2f module is more effective at capturing relationships between target features, making it particularly suitable for feature extraction in this context. The structure of the C2f module is illustrated in Figure 3.

(2) FasterNet Module. As an innovative neural network architecture, it is designed to address the limitations of computational efficiency and inference speed in traditional networks [26]. The relationship among latency, *FLOPs* and *FLOPS* are shown below:

(3) $$Latency=\frac{FLOPs}{FLOPS}$$

where *FLOPs* indicates Floating Point Operations and *FLOPS* indicates Floating Point Operations per Second. The backbone of the FasterNet architecture effectively increases detection speed by reducing the number of FLOPs while improving the FLOPS. This approach minimizes computational resource consumption while maintaining or even enhancing model accuracy [27].

Building upon the C2f module, the proposed FasterNet module replaces the standard convolution in the Bottleneck with a Pconv. This substitution significantly reduces computational cost and memory usage. Specifically, the PConv performs convolution on only one-quarter of the input channels while keeping the remaining three-quarters unchanged. These unchanged channels are then concatenated with the convolved ones at the output.

**Figure 4:**
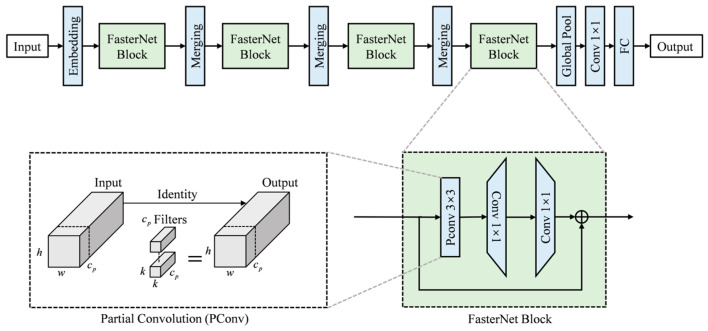
Schematic diagrams of the FasterNet modules [27]. Note: This figure presents the overall structure of FasterNet to clarify the functional context of the PConv operation. The core focus of this study is the PConv component in the bottleneck module (marked in the figure), which is the only part adopted to replace standard convolutions, reducing computational cost while preserving feature information.

This design drastically decreases both FLOPs and memory access during feature extraction and gradient flow propagation, thereby accelerating model inference and better meeting the requirements of real-time industrial inspection. It is noteworthy that the partial convolution operation preserves both the number of input channels and the spatial dimensions of the feature maps. Its objective is to minimize redundant computations while retaining information across all original channels. Although PConv in FasterNet reduces computational redundancy by operating on a subset of channels, the subsequent EMA module maintains its effectiveness. EMA is positioned to receive the full feature representation output by the backbone and employs its parallel attention branches to effectively capture multi-scale spatial and channel dependencies, ensuring that the critical features for defect detection are accurately weighted, despite the initial efficiency-driven compression by PConv.The structure of the FasterNet module is illustrated in Figure 4.

(3) EMA Mechanism. An attention mechanism module is introduced to enhance the model’s ability to extract critical feature information from terminal images. This mechanism improves the neural network’s pixel-level attention to feature maps. For insulation sleeve recognition in this study, the difference between shallow and normal insulation crimps lies solely in the variation in insulation length, with no other distinct visual features. Therefore, pixel-level attention helps the model better distinguish between these two cases.

The EMA mechanism performs feature grouping by dividing the channel dimension into multiple sub-features after the feature map is input. Each group of features is then enhanced through attention weights learned by the network [28]. EMA extracts attention weights through three parallel pathways. On one hand, features along two directions of the image channels are connected and share a 1 × 1 convolution without dimensionality reduction, enabling the two parallel branches to obtain different cross-channel interaction features. On the other hand, a separate branch omits normalization and pooling operations to capture multi-scale feature representations.

**Figure 5:**
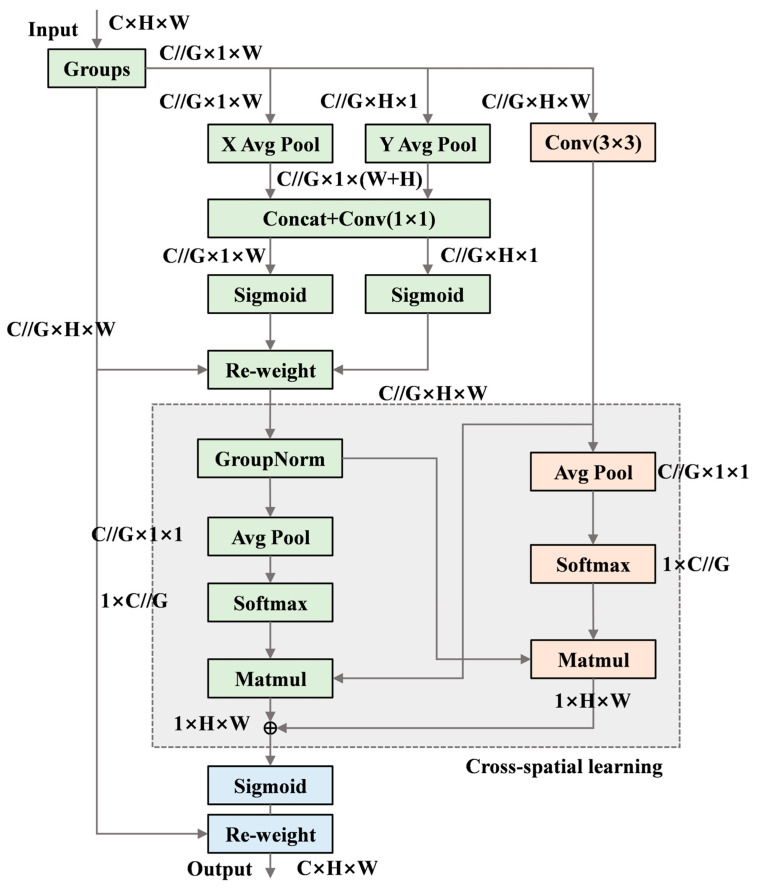
Structure of the EMA Mechanism.

Moreover, the EMA module performs cross-spatial learning, aggregating spatial information from different spatial dimensions. First, the information from the 1 × 1 convolution branch is normalized, transforming the feature channels with the smallest outputs into dimensionally consistent forms to achieve cross-spatial information aggregation [29]. Finally, a cross-spatial interaction module aggregates the attention weights from the two spatial dimensions, and the enhanced feature map is obtained through an activation function to strengthen the original features. The structure of the EMA mechanism is illustrated in Figure 5 [28].

#### 3.2.2. Improvement of the Loss Function

The bounding-box loss function measures the similarity between a predicted box and a ground-truth box. Different bounding-box losses vary in their ability to reflect this similarity, and the loss value directly affects how predicted boxes are optimized during model training; therefore, the choice of loss function must be made carefully. The basic loss function is IOU, defined as the intersection-over-union between the predicted box and the ground-truth box [30]:

(4) $$IOU=\frac{S_{T}\cap S_{P}}{S_{T}\cup S_{P}}$$

where *S_T_* represents the area of the ground-truth box, and *S_P_* represents the area of the predicted box. The intersection-over-union between the predicted and ground-truth boxes determines their degree of overlap—the higher the overlap, the greater the IoU value, indicating better model performance. However, IoU has certain limitations. Specifically, when the two boxes do not overlap, the gradient becomes zero, preventing backpropagation and thus hindering model optimization. Moreover, when IoU values are equal, the actual overlap between predicted and ground-truth boxes may still differ, meaning IoU loss cannot accurately express these discrepancies. Therefore, more advanced bounding-box loss functions are required to improve precision [31].

In this study, the SIoU bounding-box loss function is employed to measure the relationship between the predicted and ground-truth boxes in terminal crimping images. The SIoU loss comprises four components: angle cost, distance cost, shape cost, and IoU cost [32]. The loss calculation formula that considers the angle cost is expressed as follows:

(5) $$\Lambda =1-2*\sin^{2}(\arcsin(\frac{c_{h}}{\sigma })-\frac{\pi }{4})$$

where $c_{h}$ represents the height difference between the centers of the ground-truth box and the predicted box, and σ denotes the distance between the centers of the two boxes.

The distance cost considering the angle cost is expressed as:

(6) $$p_{x}={\left(\frac{b_{cx}^{T}-b_{cx}^{P}}{c_{w}}\right)}^{2}$$

(7) $$p_{y}={\left(\frac{b_{cy}^{T}-b_{cy}^{P}}{c_{h}}\right)}^{2}$$

(8) γ=2−Λ

(9) $$\Delta =\sum_{t=x,y}\left(1-e^{-\gamma p_{t}}\right)$$

where $b_{cx}^{T}$ $b_{cy}^{T}$ and denote the coordinates of the center of the ground-truth bounding box, and $b_{cx}^{P}$ and $b_{cy}^{P}$ denote the coordinates of the center of the predicted bounding box.

The shape cost is defined as follows:

(10) $$\omega_{w}=\frac{\left|w_{P}-w_{T}\right|}{\max\left(w_{P},w_{T}\right)}$$

(11) $$\omega_{h}=\frac{\left|h_{P}-h_{T}\right|}{\max\left(h_{P},h_{T}\right)}$$

(12) $$\Omega ={\sum_{t=w,h}\left(1-e^{-wt}\right)}^{\theta }$$

where $w_{P}$ and $h_{P}$ represent the width and height of the predicted bounding box, $w_{T}$ and $h_{T}$ represent the width and height of the ground-truth bounding box, and θ denotes the attention factor for the shape cost, which is set to 1 in this study.

In summary, the final formulation of the SIOU bounding box loss is expressed as follows:

(13) $$LOSS_{SIOU}=1-IOU+\frac{\Delta +\Omega }{2}$$

Since the SIOU loss incorporates the vector angle between the target regression values, it can accelerate convergence and improve regression accuracy. This enhancement allows the predicted bounding boxes for defective areas in terminal crimping to align more precisely with the ground-truth boxes, thereby enabling more accurate localization and classification of terminal crimping quality defects.

### 3.3. Experimental Setup and Evaluation Metrics

The model training in this study was completed on a Windows 11 workstation with an Intel Core i5-10700K processor (16 GB of RAM) (Intel, Santa Clara, CA, USA), and an NVIDIA GeForce RTX 3070 GPU (8 GB of VRAM) (NVIDIA, Santa Clara, CA, USA). The software environment consisted of Python 3.7, PyTorch 1.8.1, CUDA 11.1, cuDNN 8.0, and Ultralytics YOLOv5 v6.1. The model training employed the SGD optimizer with an initial learning rate of 0.01, a momentum parameter of 0.937, and a weight decay of 0.0005. The total number of training epochs was 200, the batch size was 8, and the input image resolution was uniformly 640 × 640. The training/validation/test set split ratio was 7:2:1 to ensure sufficient training and reliable evaluation.

The study employed three evaluation methods: confusion matrix analysis, precision–recall (PR) curve assessment, and loss function evaluation. The confusion matrix provides a clear view of any misclassifications of terminal crimping quality defects, enabling an assessment of classification accuracy. The precision–recall curve mitigates potential bias in accuracy estimation caused by imbalanced sample distributions. The loss function measures the discrepancy between the model’s predictions and the ground-truth values, playing a critical role in determining overall model performance. Together, these three evaluation methods provide a comprehensive assessment of the performance of TCQI-YOLOv5.

The accuracy in a confusion matrix is defined as the percentage of correctly predicted samples out of the total number of samples, as shown in Equation (14). Here, *A* represents accuracy; *TP* (true positives) denotes the number of samples correctly predicted as positive; *FN* (false negatives) denotes the number of samples incorrectly predicted as negative while actually positive; *FP* (false positives) denotes the number of samples incorrectly predicted as positive while actually negative; and *TN* (true negatives) denotes the number of samples correctly predicted as negative [33].

(14) $$A=\frac{TP+TN}{TP+TN+FP+FN}$$

Average Precision *AP* reflects the model’s accuracy in detecting individual object categories. Precision *P* quantifies the model’s classification performance on target samples, while recall *R* measures the model’s ability to localize positive samples. The *mAP* is the mean of the average precision values across all categories, where *N* denotes the total number of categories. The evaluation formulas for these metrics are expressed as follows [34]:

(15) $$P=\frac{TP}{TP+FP}$$

(16) $$R=\frac{TP}{TP+FN}$$

(17) $$AP=\int_{0}^{1}P\left(R\right)dR$$

(18) $$mAP=\frac{1}{N}\sum_{i=1}^{N}AP_{i}$$

## 4. Results

### 4.1. Performance Analysis of Various Defects

To comprehensively evaluate the TCQI-YOLOv5 model, Table 4 quantifies the detailed detection performance of various defect types. The model demonstrates strong detection capabilities across all eight defect types, with F1 scores exceeding 0.94. Notably, in the most challenging jypqd category, which has a crucial difference in determining connection quality, the model achieved a high F1 score of 0.941. This indicates a balanced performance between precision (0.963) and recall (0.920). The small gap between precision and recall suggests that while the model is highly reliable in predicting jypqd, there are still a few missed detections (possibly critical insulation length defects). Furthermore, the model performs exceptionally well in detecting subtle defects such as yjblt, achieving an F1 score of 0.971. The F1 scores for both the macro-mean and weighted average exceed 0.98, confirming the model’s overall effectiveness and stability in handling imbalanced class distributions in the dataset.

### 4.2. Confusion Matrix

**Figure 6:**
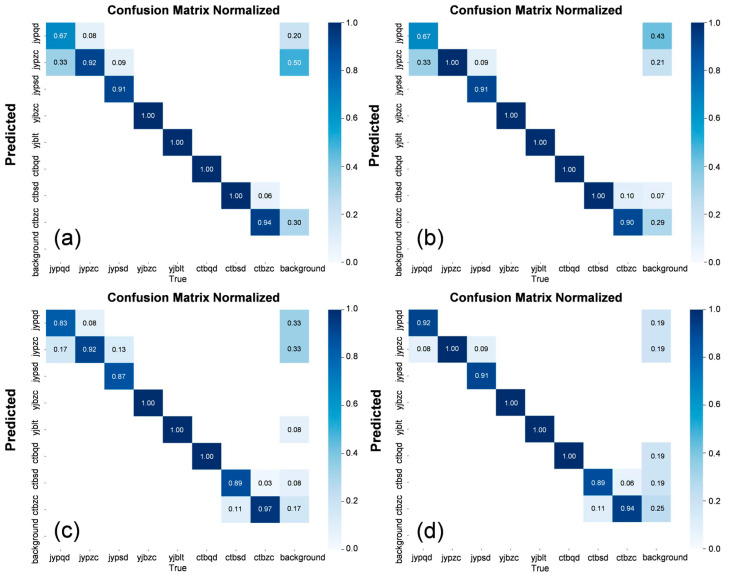
Confusion Matrices of Different Models. (**a**) represents the original model, (**b**) shows the confusion matrix after replacing the C3 module with the C2f module, (**c**) shows the matrix after changing the loss function to SIOU, and (**d**) presents the confusion matrix when both the feature extraction module and the loss function were modified simultaneously. In each confusion matrix, the columns represent the true defect types, and the rows represent the predicted defect types.

The confusion matrix is shown in Figure 6. Examining the confusion matrix of the original model, the main issues are concentrated in the insulation part, with the most frequent problem being the misclassification of shallow insulation damage as normal. When the original C3 feature extraction module was replaced with the C2f module, the detection of normal insulation improved in accuracy, but the detection of shallow insulation damage showed no significant improvement.

When the loss function was changed to SIOU, the detection accuracy for shallow insulation damage increased from 67% to 83%, a 16% improvement, with a noticeable reduction in misclassification. When both improvements were applied simultaneously, the accuracy further increased to 92%, demonstrating that the modified model substantially enhanced detection precision for defects in the insulation area. These results indicate that the proposed improvements effectively enhance the accuracy of terminal crimping quality defect detection.

### 4.3. PR Curve

**Figure 7:**
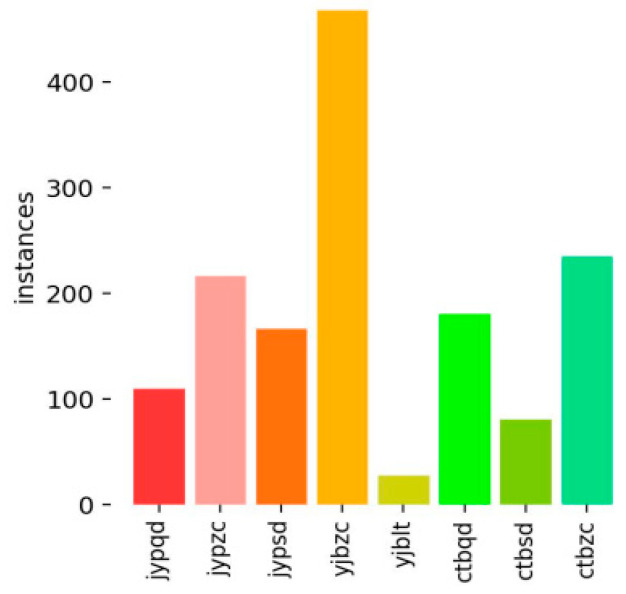
Class Distribution of TCQI (The meaning of the horizontal axis has been described in Table 3).

In cases of sample imbalance, accuracy alone is often insufficient to effectively evaluate model performance. As shown in Figure 7, the number of normal insulation samples is significantly higher than that of other defect types. If the model predicts all insulation samples as normal, it may still achieve a high accuracy, leading to misleading evaluation results. To address this, precision and recall are introduced as more reliable metrics, providing a more comprehensive assessment of the model’s ability to recognize each category.

By setting recall as the horizontal axis and precision as the vertical axis, and plotting the results as a curve, the PR curve is formed, which illustrates the relationship between precision and recall. The closer the curve is to the top-right corner, the better the predictive performance.

**Figure 8:**
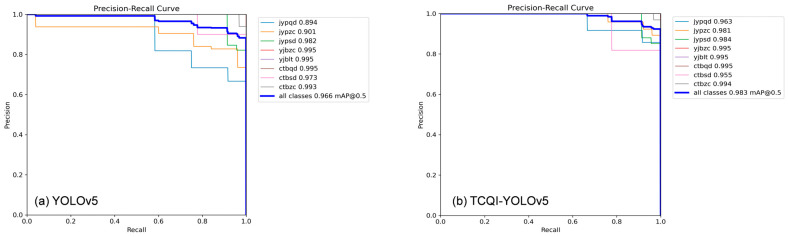
PR Curves Before and After Improvement.

As shown in Figure 8, the PR curves of the TCQI-YOLOv5 model outperform the original model for all defect types except for deep crimping at the header. The mean average precision (mAP) increased from 96.6% to 98.3%. For the critical issue of shallow insulation damage, the original precision was only 89.4%, which improved to 96.3% after the enhancements, a 6.7% increase. This indicates that the TCQI-YOLOv5 model not only significantly improves the detection and differentiation of shallow and normal insulation regions—areas that were previously challenging—but also enhances overall detection accuracy while focusing on this key region. These results demonstrate that TCQI-YOLOv5 is better suited for terminal crimping quality inspection tasks.

### 4.4. Loss Function

**Figure 9:**
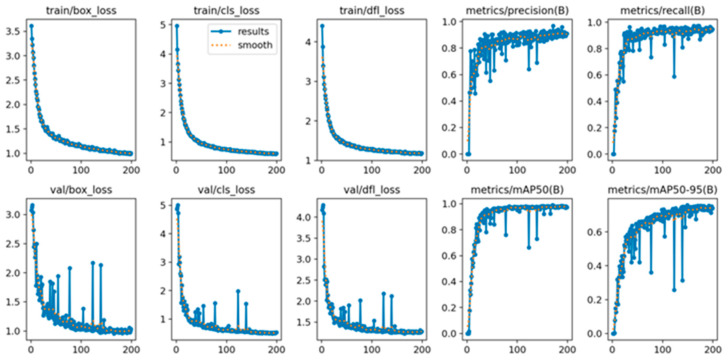
Loss Function and mAP Curves of the TCQI-YOLOv5 Model.

During model training, plotting the loss function curve allows for evaluation of the training process. The horizontal axis represents the number of iterations, and the vertical axis represents the loss value, illustrating the convergence behavior of the loss function over time. In Figure 9, the first row shows the variation in the loss on the training set across iterations, while the second row shows the corresponding variation on the test set.

From Figure 9, it can be observed that the loss decreases significantly during the first 30 iterations. By around 100 iterations, the rate of decrease begins to slow, though the loss continues to decline until the completion of 200 iterations. The continuous decrease in loss indicates that, as the number of iterations increases, the model parameters are progressively optimized, bringing the predicted values closer to the ground-truth values. The fact that the loss curve continues to decline until the end of training demonstrates the effectiveness of the model training process.

Observing the mAP curve in the figure, we can see a significant increase during the first 50 iterations. After 100 iterations, the curve continues to rise slowly and stabilizes around 0.983. The mAP curve provides a more intuitive visualization of how the model’s performance in classifying terminal crimping defects improves with increasing training iterations. The continuous upward trend of the mAP curve, ultimately maintaining a high level, further demonstrates the excellent performance of the TCQI-YOLOv5 model.

The substitution of the default IoU with the SIoU loss function yielded a statistically significant enhancement in bounding box localization accuracy. As quantified in Table 5, the model regressed boxes with a higher mean IoU (0.923 vs. 0.891) and a lower mean center-point error (2.15 px vs. 3.42 px). This performance gain is attributed to the SIoU’s comprehensive cost function, which incorporates angle and shape considerations to steer the regression optimization more effectively than the baseline.

## 5. Discussion

### 5.1. Ablation Experiment

To systematically evaluate the contribution of each component, a stepwise ablation study was conducted based on the YOLOv5 baseline model, and the results are summarized in Table 6. Replacing the original C3 module with the C2f structure improved mAP by 0.6%, verifying its enhancement of feature representation and gradient flow for small targets such as insulating sleeves. Integrating the FasterNet module further improved mAP to 97.5% while reducing the frame rate, confirming its role in improving computational efficiency through local convolution. Introducing the EMA mechanism brought the largest single-step improvement (mAP improvement of +0.4%), directly demonstrating its crucial role in enhancing pixel-level sensitivity. This mechanism can identify and distinguish subtle length changes between shallow and conventional insulating crimps. Finally, the SIoU loss function was used to optimize bounding box regression, ultimately achieving the best overall mAP value of 98.3%. It is worth noting that using SIoU alone also improved mAP by +0.5%, mainly achieved by improving localization accuracy. The incremental performance improvements, combined with the observable effects of each independent modification, clearly reveal the individual contributions and synergistic effects of the C2f structure, FasterNet module, EMA mechanism, and SIoU loss function to the outstanding performance of the final model.

### 5.2. Advantages of TCQI-YOLOv5

To further evaluate the performance of the TCQI-YOLOv5 model in identifying terminal crimping defects, a comparison was conducted among different models, with the results shown in Table 7. The Faster-RCNN model exhibited relatively poor defect classification capability, achieving a mAP of only 68.3% and low computational efficiency, with an average frame rate of 32 fps. The SSD model showed a modest improvement in accuracy, reaching 73.4%, and its main advantage was real-time performance, achieving a frame rate of 127 fps.

The YOLOv5s and YOLOv5m models demonstrated significantly higher mAP values compared to the above models, reaching 95.7% and 96.6%, respectively. The proposed TCQI-YOLOv5 model further improved the mAP to 98.3%, with a frame rate of 53 fps. The slight reduction in frame rate is attributed to the increased computational load from modifications to the feature extraction module and the loss function. Nevertheless, the detection time per image is only 0.189 s, far exceeding the requirements for practical inspection tasks. These results indicate that the base model choice is strong, and the proposed improvements further enhance overall performance.

The superior performance of the TCQI-YOLOv5 model stems from our improvements. C2f-fast reduces redundancy and maintains rich, fine-grained local features through channel grouping and residual reuse, enabling the network to more stably capture extremely subtle visual differences such as insulation length and exposed copper edges. This information typically occupies only a small pixel area in terminal images but is crucial for determining crimping quality, thus significantly improving recall and precision. The EMA mechanism adaptively amplifies and suppresses features in both channel and spatial dimensions, enhancing the network’s sensitivity to subtle differences in length, boundaries, and texture, particularly effective in distinguishing categories like “shallow crimp” and “normal,” which only exhibit length differences. PConv significantly reduces FLOPs and memory usage while maintaining key feature representation capabilities, thereby improving inference throughput (FPS) and facilitating real-time deployment and engineering implementation in industrial production lines. SIOU incorporates angle, distance, and shape into the regression metrics, more accurately constraining elongated or slightly offset prediction boxes compared to IOU/CIoU, reducing localization errors for small targets and thus decreasing false negatives and missed detections.

Leveraging the advantages of the TCQI-YOLOv5 model, this system is suitable for scenarios requiring a balance between accuracy and real-time performance, such as continuous inspection stations for automotive wiring harnesses. TCQI-YOLOv5 can effectively identify terminals with visual differences primarily manifested in length and boundaries, such as shallow/deep insulation, abnormal lead length, and exposed copper on the pressure feet—terminal types distinguished by geometric and boundary features. Furthermore, the model performs optimally in stations with relatively stable camera angles and lighting designs.

### 5.3. Limitations and Future Prospects

Although TCQI-YOLOv5 performs well on the current dataset, it still has several limitations that require further research and improvement in future work. The limitations of TCQI-YOLOv5 are primarily categorized into two areas. The first type is challenges with extremely small or blurred defects. The model sometimes fails to detect minute defects, particularly “terminal burrs” or “slight copper exposure” when they are located near edges or appear blurred due to motion, complex lighting conditions, or low-resolution imaging. This limitation stems from the down sampling operations in the backbone network, which cause the loss of fine-grained spatial information necessary for distinguishing these tiny features from the background. The second type is inter-class confusion in complex backgrounds. In a few instances, the model incorrectly classifies a defect that shares structural similarities with another class. For example, slight variations in the background texture or wire strands are sometimes misclassified as “wire exposure,” leading to false alarms. While the C2f-fast-EMA module enhances feature fusion, the distinction between highly similar defect types in noisy backgrounds remains a challenge, suggesting that the model needs more discriminative power for subtle inter-class differences.

To address these limitations, we will explore High-Resolution Feature Fusion techniques, such as integrating multi-scale feature maps at an earlier stage, to better preserve the location and detail of small targets. Furthermore, training can incorporate images of various terminal morphologies, allowing terminals to be categorized into several major types. Terminal type can be determined first based on appearance, and then crimping quality detection can be achieved based on the type and location of different crimping quality issues. Future work will consider integrating newer versions of YOLO as more advanced versions are released to further improve detection performance. Exploring these models may provide additional insights for optimizing our current method.

## 6. Conclusions

This study successfully proposed and validated an improved TCQI-YOLOv5 model, providing an efficient and reliable automated solution to address the challenges of insufficient accuracy and low efficiency in terminal crimping quality inspection. The main contributions and conclusions of this study can be summarized in three aspects:

1. Model Architecture Optimization: By replacing the original C3 module with the C2f-fast-EMA module, the network’s feature extraction capability was significantly enhanced, particularly in capturing subtle length differences in insulation areas. Additionally, the introduction of the FasterNet module improved computational efficiency while maintaining high accuracy.
2. Loss Function Improvement: The SIOU loss function was adopted to replace the traditional IOU loss. By comprehensively considering the angle, distance, and shape costs between bounding boxes, the localization accuracy of predicted boxes was significantly improved, enabling more precise detection of defect areas.
3. Significant Performance Enhancement: Experimental results demonstrate that the improved model achieved an overall mAP of 98.3%. Notably, for the challenging defect of shallow insulation damage, precision increased markedly from 89.4% to 96.3%. The detection speed also fully meets the real-time requirements of industrial production lines.

The TCQI-YOLOv5 model exhibits excellent overall performance in terminal crimping quality inspection, validating the effectiveness of the proposed improvements. This study provides valuable insights for precise defect detection in industrial applications. Future work will focus on enhancing the model’s generalization to terminals of various shapes and integrating more advanced detection algorithms to further improve performance.

## Figures and Tables

**Table 1:** Causes and Morphologies of Defects in the Three Terminal Areas.

Defect Type	Cause of Defect	Morphology of Defect
Deep Insulation Crimping, Shallow/Normal Wire Tip	Insufficient Wire Stripping	Connection Between Insulation Sleeve and Crimp Barrel, Wire Tip Exposed Too Short or Normal
Deep Insulation Crimping, Deep Wire Tip Crimping	Conductor Misalignment	Connection Between Insulation Sleeve and Crimp Barrel, Wire Tip Overexposed
Normal Insulation Crimping, Deep Wire Tip Crimping	Excessive Stripping or Conductor Cut	Wire Tip Overexposed
Normal Insulation Crimping, Shallow Wire Tip Crimping	Insufficient Stripping	Wire Tip Underexposed
Shallow Insulation Crimping, Deep/Normal Wire Tip Crimping	Excessive Stripping	Insulation Not Exposed, Wire Tip Overexposed or Normal
Shallow Insulation Crimping, Shallow Wire Tip Crimping	Conductor Misalignment	Insulation Not Exposed, Wire Tip Underexposed
Copper Exposure at Crimp Barrel	Loose Crimping	Conductor Exposed at Crimp Barrel

**Table 2:** Hikvision MV-CU060-10GC Camera Parameters.

Parameter Name	Parameter Value
Resolution	3072 × 2048
Frame Rate	19.1 fps
Pixel Size	2.4 μm × 2.4 μm
Data Interface	GigE (1000 Mbit/s)
Dimensions	29 mm × 29 mm × 42 mm

**Table 3:** TCQI Categories and Their Corresponding Labels.

TCQI Categories	Class Labels
Shallow Insulation Crimping	jypqd
Normal Insulation Crimping	jypzc
Deep Insulation Crimping	jypsd
Normal Crimp Barrel	yjbzc
Copper Exposure at Crimp Barrel	yjblt
Shallow Wire Tip Crimping	ctbqd
Deep Wire Tip Crimping	ctbsd
Normal Wire Tip Crimping	ctbzc

**Table 4:** Class-by-class performance metrics of the TCQI-YOLOv5 model on the test set.

Class Labels	Accuracy	Recall Rate	F1 Score
jypqd	0.963	0.920	0.941
jypzc	0.991	0.985	0.988
jypsd	0.992	0.990	0.991
yjbzc	0.994	0.998	0.996
yjblt	0.978	0.965	0.971
ctbqd	0.985	0.980	0.982
ctbsd	0.990	0.975	0.982
ctbzc	0.993	0.995	0.994
Macro average	0.985	0.976	0.98
Weighted average	0.987	0.983	0.985

**Table 5:** Comparison of localization accuracy between IoU and SloU loss functions.

Loss Function	Average IoU	Average SIoU	Center Point Average Error
CIoU	0.891	127	3.42
SIoU	0.923	32	2.15

**Table 6:** Comparison of Results Across Ablation Experiment.

Experiment Number	Model	mAP@0.5 (%)	FPS	mAP Improvement
1	YOLOv5	96.6	87	-
2	YOLOv5 + C2f	97.2	85	+0.6
3	YOLOv5 + C2f + FasterNet	97.5	79	+0.9
4	YOLOv5 + C2f + FasterNet + EMA	97.9	61	+1.3
5	YOLOv5 + C2f + FasterNet + EMA + SIoU	98.3	53	+1.7
6	YOLOv5 + SIoU	97.1	86	+0.5

**Table 7:** Comparison of Results Across Different Models.

Model	mAP	FPS
SSD [35]	73.4%	127
Faster-RCNN [36]	68.3%	32
YOLOv5s [37]	95.7%	108
YOLOv5m [37]	96.6%	87
TCQI-YOLOv5 (Proposed Model)	98.3%	53

## Data Availability

The original contributions presented in this study are included in the article. Further inquiries can be directed to the corresponding author.
